# IDENTIFICATION AND MACHINE LEARNING PREDICTION OF MEDICARE BENEFICIARIES’ COGNITIVE FUNCTION TRAJECTORIES

**DOI:** 10.1093/geroni/igae098.2182

**Published:** 2024-12-31

**Authors:** Haiqun Lin, Weiyi Xia, Anum Zafar, Hyosin Kim, Soko Setoguchi, Fred Kobylarz, Jason Roy, Olga Jarrín

**Affiliations:** Rutgers, The State University of New Jersey, Newark, New Jersey, United States; Rutgers, The State University of New Jersey, Piscataway, New Jersey, United States; Rutgers, The State University of New Jersey, New Brunswick, New Jersey, United States; Oregon State University, Corvallis, Oregon, United States; Rutgers, The State University of New Jersey, New Brunswick, New Jersey, United States; Rutgers Robert Wood Johnson Medical School, New Brunswick, New Jersey, United States; Rutgers, The State University of New Jersey, Piscataway, New Jersey, United States; Rutgers, The State University of New Jersey, New Brunswick, New Jersey, United States

## Abstract

This work aims to characterize the functional decline in cognition during the last five years of life. A 10% random sample consisting of 195,240 Medicare beneficiaries who died in 2019, aged fifty or older and continuously enrolled in Medicare during their last five years of life, was analyzed. Cognitive function status, characterized by no impairment, memory loss, mild cognitive impairment, and dementia, was obtained from the assessment and the claim file for each beneficiary whenever it was available. Changes in cognitive function over the last five years were examined using the group-based trajectory model for ordered categorical outcomes: Ten trajectory groups of cognitive function were identified and had a better value of Bayesian Information Criterion than either the 9-class or the 11-class solution. The 10 trajectory groups were categorized into four clusters: the cognitively intact cluster (38% of beneficiaries) which contains one trajectory group with beneficiaries maintaining their cognitive function throughout the last five years, the mild cognitive impairment (MCI) cluster (27%) which contains four trajectory groups with beneficiaries experiencing slow decline in cognitive function and died with MCI, the steady decline cluster to dementia (25%) which contains four trajectory groups with beneficiaries experiencing steady decline in cognitive function and died with dementia, and the early-decline dementia cluster (10%) which contains only one trajectory group with beneficiaries whose cognitive decline started more than five years before death. Sociodemographic and clinical factors were assessed for their association with the trajectories and used to predict trajectory clusters with machine learning algorithms.

